# Genetic and biotechnological potential of thermophilic *Streptomyces* sp. isolated from Baikal freshwater psychrophilic sponge

**DOI:** 10.1038/s41598-025-25364-y

**Published:** 2025-11-21

**Authors:** Maria Dmitrieva, Victoria Shelkovnikova, Maria Morgunova, Ekaterina Malygina, Natalia Imidoeva, Alexander Belyshenko, Tamara Telnova, Tatyana Vavilina, Alexander Konovalov, Anna Batalova, Olga Lipatova, Angelika Listopad, Denis Axenov-Gribanov

**Affiliations:** Laboratory of Experimental Neurophysiology, Irkutsk State University, Irkutsk, Russia 664003

**Keywords:** Natural products, Mass spectrometry, Biotechnology, Ecology, Freshwater ecology

## Abstract

Microorganisms inhabiting extreme environmental conditions receive special attention because they possess different adaptations to adverse conditions. Currently, their biotechnological potential and ability to isolate biologically active metabolites have increased. The increasing mortality due to different diseases has become particularly important as one of the notable challenges in modern healthcare. This highlights the necessity of discovering new producers of natural products (NPs). The aim of this study was to evaluate the genetic and biotechnological potential through the assessment of NP synthesis and genome annotation of the thermophilic strain *Streptomyces* sp. LPB2020-019-1HS. The thermophilic strain was isolated from the Baikal endemic cold water sponge *Lubomirskia baikalensis*. Subsequently, *Streptomyces* sp. LPB2020-019-1HS was cultivated at six temperatures ($13\,^\circ$C, $28\,^\circ$C, $37\,^\circ$C, $45\,^\circ$C, $55\,^\circ$C, and $65\,^\circ$C) in twelve nutrient media with different compositions (nutrient rich and nutrient poor). Using high-performance liquid chromatography and mass spectrometry approaches, the synthesis of compounds by the strain was assessed at $13\,^\circ$C, $28\,^\circ$C, and $37\,^\circ$C. Antimicrobial activity was evaluated at all temperatures (from $13$ to $65\,^\circ$C). We demonstrated the presence of antibiotic activity against *Bacillus subtilis* for strains cultivated at 28 °C, $37\,^\circ$C, and $45\,^\circ$C. Additionally, we observed activity against *Mycobacterium smegmatis* when the strain was cultivated at $28\,^\circ$C, $37\,^\circ$C, $45\,^\circ$C, and $55\,^\circ$C. Furthermore, the strain exhibited activity against *Escherichia coli*, *Pseudomonas putida*, and *Candida glabrata* when cultured at $37\,^\circ$C. Overall, we found that *Streptomyces* sp. LPB2020-019-1HS produces a family of NPs related to Nocardamine and hypothesized that freshwater Actinobacteria have mechanisms for chelating iron ions, making them available for plants/sponges or other symbiotic organisms. Therefore, our research findings underscore the importance of studying extremophilic microorganisms from Lake Baikal in the context of developing new pharmaceuticals and biotechnological solutions for contemporary healthcare challenges.

## Introduction

The global healthcare crisis represents one of the most pressing and important challenges facing the global community^1^. This crisis is associated with a multitude of challenges, including threats to public health, disparities in access to medical care, and the resilience of healthcare systems in the face of extreme pressures, such as epidemics and pandemics^2,3^. One of the great problems of modern healthcare is the increase in population mortality from diseases caused by antibiotic-resistant strains of microorganisms^4,5^. The development of standard treatments for infectious diseases is rapidly losing relevance due to the discontinuation of medications that have become ineffective in clinical practice^6,7^. Some drugs currently used as therapeutic agents are derived from natural sources^8,9^. Recently, special attention has been paid to extremophilic microorganisms, as they serve as factories for producing highly efficient NP^10–13^. These microorganisms live in extreme environments, such as deep-sea hydrothermal vents, acid hot springs, or frozen Arctic regions, and have proven to be rich sources of novel bioactive molecules with outstanding medicinal properties^14,15^. Their ability to live in extreme conditions has forced them to develop extraordinary adaptations, which often involve the production of NPs. These compounds have promising antimicrobial, anticancer, and anti-inflammatory properties^15^. The use of extremophilic strains of actinobacteria is one of the most promising strategies, as actinobacteria synthesize diverse compounds and are widely distributed in different stress environments^12^. Therefore, antibacterial drugs have been developed for the treatment of infectious diseases^16^. Several notable examples of such drugs include lefamulin, plitidepsin, salinosporamide A, and daptomycin. Lefamulin is a semisynthetic pleuromutilin antibiotic derived from natural compounds produced by *Streptomyces* species, which are well known for their resilience in extreme environments. This drug has been implemented in clinical practice and is widely used for the treatment of respiratory tract infections caused by multidrug-resistant bacteria^17^. Plitidepsin, which was originally derived from marine actinobacteria, has shown significant antitumor and antiviral properties^18^. During the COVID-19 pandemic, this drug has also been in clinical trials for the treatment of coronavirus infection^19^. Actinobacteria are gram-positive bacteria and are characterized by a high (approximately 60%) content of guanine and cytosine in their DNA^20^. The vital activities of actinobacteria are directly influenced by different physicochemical parameters, such as hydrostatic pressure, temperature fluctuations, oxygen levels, and pH values^21,22^. They live in diverse environments, and their metabolic capabilities are influenced by the specific conditions of their surroundings. For example, in thermophilic actinobacteria, enzymes and proteins exhibit high thermostability, preventing their denaturation at extreme temperatures^23^. The proteins of these bacteria have a greater number of hydrogen bonds, disulfide bridges, and other intramolecular interactions. The membranes of thermophilic actinobacteria contain saturated fatty acids, which ensure membrane stability^24^. Psychrophilic actinobacteria live in low-temperature environments. Their key adaptations include antifreeze proteins and unsaturated fatty acids^25^. Proteins bind to ice crystals, preventing damage to cellular structures during freezing. A high content of unsaturated fatty acids in the membranes allows them to maintain fluidity at low temperatures^25^. Halophilic actinobacteria are capable of surviving in environments with high salt concentration^26^. These bacteria synthesize special molecules, such as glycine betaine, which help maintain osmotic balance within the cell, preventing dehydration^27^. Some actinobacteria can survive under high-radiation conditions. These bacteria have developed mechanisms for the rapid and effective repair of DNA damage caused by radiation^28^. Additionally, these microorganisms synthesize antioxidants that protect cells from oxidative stress^29^. In this way, extremophilic actinobacteria, as part of their biochemical adaptations, synthesize many NPs^30^. These compounds often include secondary metabolites, such as antibiotics, enzymes, and other bioactive molecules, which not only contribute to their survival in extreme environments but also hold significant potential for various biotechnological and pharmaceutical applications^30,31^. Special attention has been given to studying thermophilic microorganisms in psychrobiotic environments, such as lake Vostok environments, soil environments in Northern Ireland and Bolivia, and extraterrestrial environments. The microbial life of microorganisms found in Lake Vostok, located beneath Antarctic ice, includes the facultative chemolithoautotroph *Hydrogenophilus thermoluteolus*, a nonspore-forming thermophile that has also been isolated from hot springs. Thermophilic and thermotolerant chemolithoautotrophs, which utilize inorganic energy sources such as hydrogen, nitrogen, sulfur, or iron to sustain their metabolism, were recently discovered in accretion ice samples taken from the inundation zone of Lake Vostok^32^. A recent study conducted via cultivation methods and 16S rRNA gene sequencing revealed the presence of cultivable thermophiles along an extreme temperature gradient on Deception Island volcano. Thermophilic representatives from the genera *Geobacillus*, *Brevibacillus*, *Anoxybacillus*, and *Thermus* and the order *Bacillales* were isolated from both fumaroles and glaciers, where temperatures ranged from $80$ to $0\,^\circ$C. Most of the thermophilic isolates found in the glaciers of Deception Island at $0\,^\circ$C were spore-forming bacteria with strong cultivation capabilities and notable resistance to desiccation and ultraviolet radiation^32,33^. Lake Baikal is an extraordinary ecosystem of the planet with great psychrobiotic potential. A lake is characterized by specific conditions, including low water temperature, low mineral and organic contents, high oxygen levels throughout its water column, and an incredibly high biodiversity of flora and fauna^34–38^. More than 2,500 animal species, most of which are endemic, are adapted to live at $4-6\,^\circ$C. This leads to narrow thermal windows and specific adaptations to cold-temperature environments. Despite the many microbiological studies performed in the ecosystem of Lake Baikal, special attention has been given to studying the microbial communities of Lake Baikal, which remain understudied^39–43^. Special interest has been given to endemic cold water organisms involved in the clearance of Lake Baikal water. These are Baikal freshwater sponges. As active biological filters and biomarkers of thermal stress and chemical pollution, Lake Baikal sponges can accumulate inside themselves as different microorganisms, forming a new environmental zone for symbionts at different levels^44,45^. Actinobacteria are sponges associated with microbial communities. Lake Baikal sponges are well known to contain cultivable and noncultivable actinobacteria. According to Parfenova et al. (2008), the genus *Micromonospora* is dominant in all Baikal sponges studied, accounting for 68% of all actinobacteria in *L. baicalensis*, 69% in *B. intermedia*, and 90% in *B. baicalensis*^46^. Additionally, as described in Kaluzhnaya et al. (2021), 17 strains of actinobacteria have been isolated from *L. baicalensis*^47^. However, only a few studies have described the extremophilic nature and genetic potential of Baikal symbiotic Actinobacteria^48^. Thus, the survival of thermophiles in a psychobiotic environment offers a new perspective on the physiological and molecular mechanisms underlying the natural cryopreservation of microorganisms. Thermophile cultures maintained at low temperatures can serve as a laboratory model for further study of the metabolic potential of thermophiles at psychrobiotic temperatures, as well as for elucidating the molecular mechanisms underlying natural preservation and adaptation to a psychrobiotic environment^32^. Owing to the numerous findings of thermophilic microorganisms in cold-water ecosystems, the hypothesis of this study was that thermophilic microorganisms were present in the filtrating cold-water organisms of Lake Baikal. Thus, the aim of this study was to evaluate the antimicrobial activity and genetic potential of the thermophilic strain of *Streptomyces *sp. isolated from Baikal cold-water endemic sponges for the synthesis of NPs*.*

## Materials and methods

### Sampling, location, and isolation of actinobacteria

Sampling of the Baikal sponge *Lubomirskia baikalensis* (Pallas, 1773) was conducted in the settlement of Listvyanka at depths of 10 m in February 2020. Two grams of sponge were immediately placed in a sterile 5 ml Eppendorf tube. In the laboratory, *L. baikalensis* samples were frozen and stored at $-86\,^\circ$C before strain isolation. According to general recommendations related to the isolation of rare actinobacteria, to isolate thermophilic strains of Actinobacteria, pretreatment related to preheating samples at $110 \,^\circ$C for 2 hours was used on a TS-100C thermoshaker (Biosan, Riga, Latvia)^49–51^. Prior to pretreatment, the glycerol where the samples were stored was changed. Glycerol was used as a negative control. Thus, the samples of *L. baikalensis* consisted of freshly prepared sterile glycerol, and the sponges were homogenized via a glass stick. The isolation and cultivation of rare actinobacterial strains were performed with several solid nutrient media, such as MS nutrient media (soy flour, d-mannitol, agar, pH 7.2), Czapek agar (NaNO3, starch, MgSO4$\times$7H2O, KCl, FeSO4$\times$7H2O, K2HPO4, agar, pH 7.2), and R2A nutrient media (yeast extract, lactose peptone, casein hydrolysate, glucose, starch, K2HPO4, anhydrous MgSO?, sodium pyruvate, agar, pH 7.2). The isolation of microorganisms was carried out at $28\,^\circ$C and $37 \,^\circ$C, and the nutrient medium was supplemented with the antibiotics cycloheximide (50 mcg/mL) and phosphomycin (100 mcg/mL )^52^. Ventilated Petri dishes with a diameter of 90 mm were used. The homogenates were not diluted and were loaded on Petri dishes in a volume of 100 mcL. The experiment was performed 3 times. The plates were incubated for 14 days and checked every 24 h for the appearance of actinobacterial colonies. The following bacteria were recognized on the basis of colony morphology: solid density of colonies, growth inside the agar media, and steady border of colonies^52^. Colonies with a leathery texture with or without aerial hyphae were transferred from the primary plates onto new MS media. Thus, only one strain related to Actinobacteria was isolated. The strain was isolated from plates with MS nutrient media at $37\,^\circ$C.

### Cultivation of actinobacteria

The *Streptomyces* sp. LPB2020-019-1HS strain was cultivated in 12 different liquid nutrient media, namely, SM1, NL19, SM17, SM12, SM20, SM24 SM25, SM27Ac, SM27N, SM27Al, R2 and minimal medium (MM), because the media composition is a determining factor for the production of secondary metabolites^53^. The composition of the nutrient media used was as follows: SM1 (soy four, glucose, Na_2_SO_4_, pH 7.0), NL19 (soy flour, D-mannitol), SM17 (soy four, glucose, glycerol, soluble starch, peptone, yeast extract, NaCl, CaCO_3_, pH 6.4), SM12 (soy four, glucose, peptone, meat extract, yeast extract, NaCl, CaCO_3_ pH 7.6), SM20 (maltose, peptone, meat extract, yeast extract, MgSO_4_ $\times$ 7 H_2_O, NaCl, pH 7.2), SM24 (yeast extract, peptone, glucose, KH_2_PO_4_, MgSO_4_$\times$7 H_2_O, pH 6.2), SM25 (peptone, malt extract, glycerol, pH 6.5), SM27Ac (soy four, glucose, peptone, meat extract, yeast extract, NaCl, CaCO_3_, pH 4.5), SM27N (pH 6.8–7.0), SM27Al (SM27Ac at pH 4.5 and pH 8.7, respectively), R2 (malt extract, yeast extract, glucose, artificial sea water, pH 7.8), MM (L-asparagine, K_2_HPO_4_, MgSO_4_$\times$7 H_2_O, FeSO_4_$\times$7 H_2_O, glucose, pH 7.2). Cultivation was carried out for 14 days at $13\,^\circ$C, 7 days at $28\,^\circ$C and 5 days at $37\,^\circ$C, $45\,^\circ$C, $55\,^\circ$C and $65\,^\circ$C. Cultivation at $13-37\,^\circ$C was conducted in 250 ml Erlenmeyer flasks with the addition of 30–50 ml of nutrient medium in an orbital shaker DOS-20 L (Elmi, Latvia, Riga) with a shaking rate of 170 rpm. Cultivation at $45-65\,^\circ$C was conducted in 500 ml Erlenmeyer flasks with the addition of 100–150 ml of nutrient medium in an orbital shaker DOS-20 L (Elmi, Latvia, Riga) with a shaking rate of 170 rpm.

### Extraction of secondary metabolites

For metabolite extraction, the strain *Streptomyces sp.* LPB2020-019-1HS was cultivated in 1 L of production medium under the specified conditions. Metabolites were extracted with an equal volume of ethyl acetate (Vecton, Russia, Saint-Petersburg)^54^. The resulting mixture was sonicated for 15 min and stirred for 1 h followed by centrifugation at 3000 rpm for 10 min^55^. The ethyl acetate were dried on and the crude extracts obtained were dissolved in methanol (Vecton, Russia, Saint-Petersburg) at a concentration of 10 mg/mL.

### Antibiotic assay of extracts

The antibiotic activity was tested using the disk diffusion method^56^. Six strains of microorganisms, *Bacillus subtilis* ATCC 66337, *Staphylococcus carnosus* ATCC51365, *Mycobacterium smegmatis* AC-1339, *Escherichia coli* ATCC 25922, *Pseudomonas putida* KT 2440, and *Candida glabrata* Y-2824, were chosen as model test cultures. For antimicrobial testing, overnight cultures of *Streptomyces sp.* LPB2020-019-1HS were grown under the described conditions and adjusted to an optical density of OD_600_ = 0.1 ± 0.02 using a spectrophotometer PE-5300V (PromEkoLab, Russia, Saint-Petersburg) (600 nm). This value corresponded to approximately 100 million CFU per milliliter according to laboratory calibration. The suspension was then diluted 1:100 in sterile saline to yield a final inoculum of about 1 million CFU per milliliter, which was used for agar surface inoculation in disk diffusion assays. Overnight *Bacillus subtilis*, *Staphylococcus carnosus* and *Pseudomonas putida* were inoculated on solid nutrient LB media (tryptone, yeast extract, NaCl), *Mycobacterium smegmatis* was inoculated on IPS media (malt extract, yeast extract, starch, CaCO_3_, pH – 7.5), and *Candida glabrata* was inoculated on PDS media (glucose, peptone, yeast extract) and dried at room temperature. Then 40 mcL of each extract was loaded onto 6-mm paper disks and the plates were incubated at $37 \,^\circ$ C for 24 h. The zones of inhibition were measured manually with an accuracy of ±1 mm^57^.

### Liquid chromatography–mass spectrometry (LC–MS) and dereplication analysis

The biotechnological potential of the strains was assessed using high-performance liquid chromatography (HPLC) on an Ultimate 3000 chromatograph (Dionex, Waltham, MA, USA) coupled with an LTQ Orbitrap high-resolution mass spectrometer (Thermo Fisher Scientific, Waltham, MA, USA). A linear gradient of solvents (water: acetonitrile) was used from 5% to 95% for separation for 18 min with a C18 column (Acquity UPLC BEH, Framingham, MA, USA) of 130 Å, 1.7 $\upmu$m, and 2.1 mm $\times$ 100 mm^53^. Mass detection was performed in positive mode, with the detection range set to 160–2500 m/z. This part of the study was conducted at Saarland University. Data were collected and analyzed using the Xcalibur software, version 3.0 (Thermo Fisher Scientific, USA). The identification of secondary metabolites was carried out via the Dictionary of Natural Products database (CRC-press. v.10.1 2019) with search parameters for accurate molecular mass (MM) and biological source of NP isolation - * streptomyces *^58^.

**Genome sequencing, assembly and annotation**

The genome was sequenced with an Illumina MiSeq platform using 250 bp paired-end reads. According to the FastQC report, the usual read length was 250 bp, while there were very few reads with lengths of 35 bp and 251 bp. For *de novo* genome assembly, we used Unicycler (v. 0.5.0)^59^ with default parameters. Table S1 shows the length and depth of each of the assembled contigs. We checked the contigs for possible contamination by performing blastn procedures against the whole NCBI database. We set a threshold for e-values of 1e^−20^ to leave contigs that strongly relate to the *Streptomyces* genus. Additionally, we checked the GC content to leave contigs with the expected GC ratio for this genus and kept contigs longer than 1000 bp. Thus, 70 contigs survived after the filtering procedure. Platanus (v. 1.2.4) was subsequently used to generate the scaffold assembly and improve it, but it was not useful and did not generate longer scaffolds. We checked assembly completeness via the BUSCO (v. 5.4.7)^60^ and streptomycetales_odb10 datasets (Table 1).

**Table 1:** BUSCO annotation result.

Number	Percent (%)	Description
1550	98.10	Complete BUSCOs (C)
1548	98.00	Complete and single-copy BUSCOs (S)
2	0.10	Complete and duplicated BUSCOs (D)
3	0.20	Fragmented BUSCOs (F)
26	1.70	Missing BUSCOs (M)
1579	100	Total BUSCO groups searched

The GC content, N50 and other assembly metrics were calculated with QUAST (v. 5.2.0)^61^ (Table2).

**Table 2:** Characteristics of genome from QUAST. By default, all measures (19-24 lines) were calculated for contigs from 500bp and longer.

Contigs	70
Contigs (>= 0 bp)	132
Contigs (>= 1000 bp)	70
Contigs (>= 5000 bp)	61
Contigs (>= 10000 bp)	51
Contigs (>= 25000 bp)	40
Contigs (>= 50000 bp)	36
Largest contig	492585
Total length	5931592
Total length (>= 0 bp)	5953768
Total length (>= 1000 bp)	5931592
Total length (>= 5000 bp)	5916638
Total length (>= 10000 bp)	5845643
Total length (>= 25000 bp)	5657079
Total length (>= 50000 bp)	5499603
For contigs >= 1000 bp	
N50	169319
N90	58991
auN	211520
L50	11
L90	34
GC (%)	72.57

The genome was annotated with Prodigal (v. 2.6.3)^62^, and the functional annotation was performed with the eggNOG-mapper web server (v. 2.1.9)^63^. The potential of the strain for the synthesis of secondary metabolites was analyzed by antiSMASH v.8.0.4^64^. Only clusters with high Similarity Confidence were included in the analysis.

### Phylogenetic tree construction

A phylogenetic tree was constructed through the 16S rRNA gene sequence obtained during genome assembly. The sequence was deposited in GenBank (NCBI sequence ID OR643632). Evolutionary history was inferred using the Neighbor-Joining tree^65^. The nucleotide sequences of the experiment were aligned with sequences with the greatest similarity from the NCBI database. Evolutionary distances were computed using the Tamura-Nei method^66^. The evolutionary analysis was conducted using MEGA X^67^.

## Results

### Strain identification

**Fig. 1:**
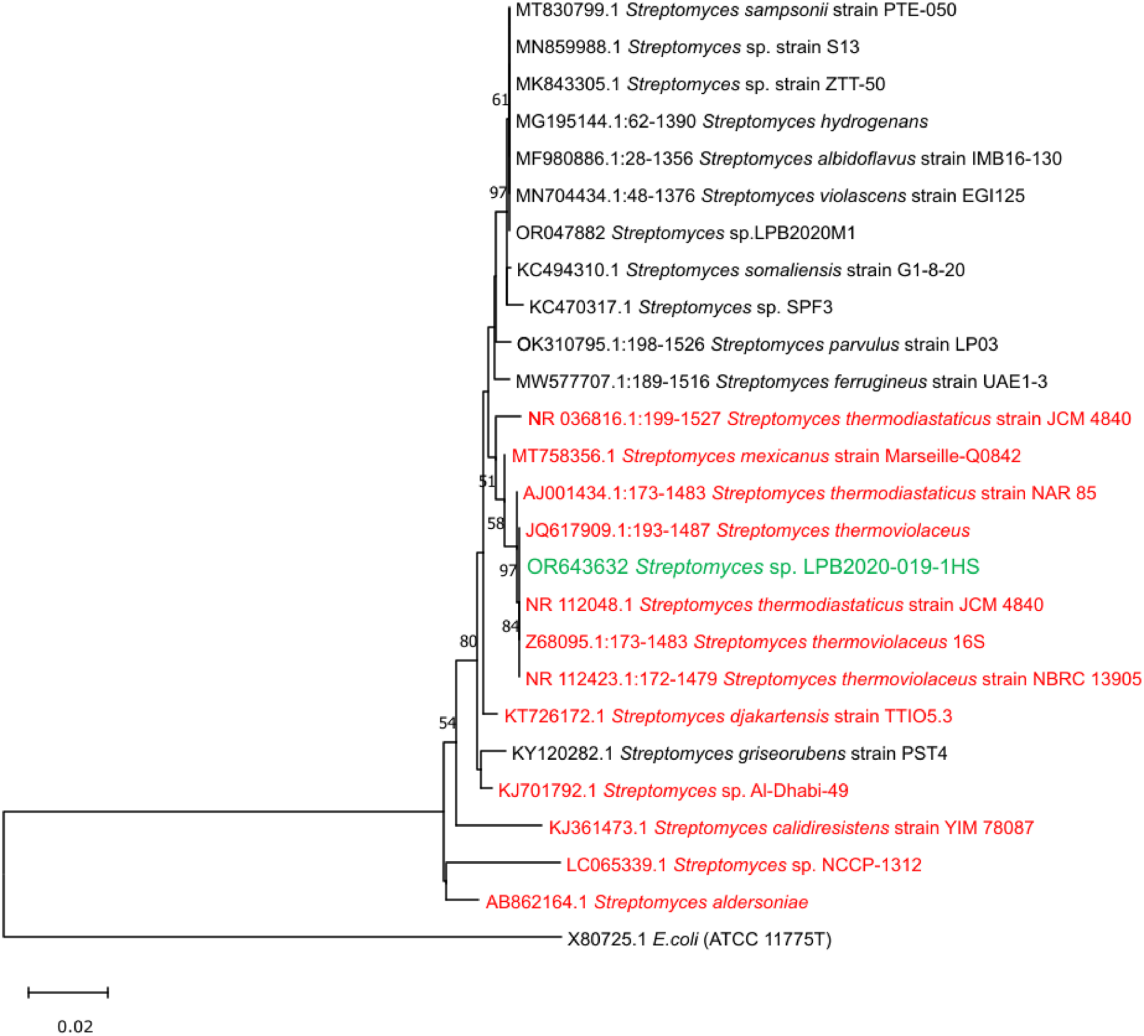
Evolutionary relationships of the strain *Streptomyces* sp. LPB2020-019-1HS. The thermophilic strains are highlighted in red. The strain *Streptomyces* sp. LPB2020-019-1HS is highlighted in green.

The strain *Streptomyces* sp. LPB2020-019-1HS was identified using 16S rRNA gene sequencing. Based on GenBank data, the strain is characterized by a high similarity to *Streptomyces thermoviolaceus* (percent identity (PI) 100%), *Streptomyces thermodiastaticus* strain JCM 4840 (PI 99.92%) and *Streptomyces mexicanus* strain Marseille-Q0842 (PI 99.62%). Figure 1 shows the phylogenetic tree of the strain *Streptomyces* sp. LPB2020-019-1HS. The phylogenetic tree confirmed that our strain has a high degree of relatedness to other thermophilic strains. The 16S rRNA gene sequence of *Streptomyces* sp. LPB2020-019-1HS was analyzed using the EzBioCloud database^68^. The analysis confirmed a high similarity of the strain to thermophilic representatives of the genus *Streptomyces*. The closest type strain was identified as *Streptomyces thermoviolaceus* subsp. *apingens* JCM 4312 with 100% sequence identity, followed by *Streptomyces thermodiastaticus* JCM 4840 and *Streptomyces thermoviolaceus* NBRC 13905, each also showing 99.9–100% identity (Fig. 1).

**Fig. 2:**
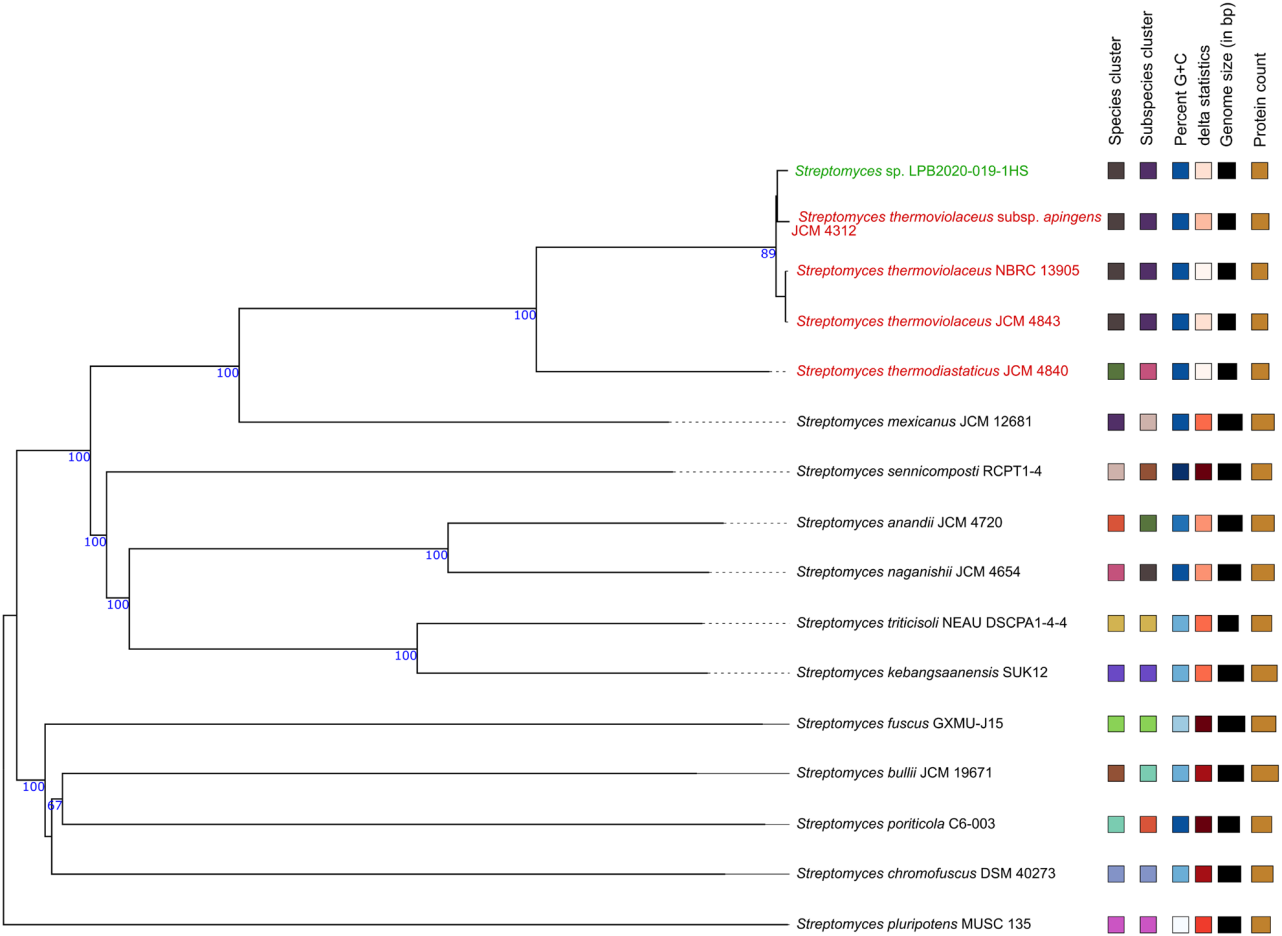
Phylogenetic tree showing the relationship between *Streptomyces* sp. LPB2020-019-1HS and related thermophilic Streptomyces strains based on whole-genome sequencing data. The thermophilic strains are highlighted in red. The strain *Streptomyces* sp. LPB2020-019-1HS is highlighted in green.

A phylogenetic tree constructed from whole-genome sequence data (Fig. 2) shows that *Streptomyces sp.* LPB2020-019-1HS clusters closely with several thermophilic representatives of the genus, including *Streptomyces thermoviolaceus* subsp. *apingens* JCM 4312, *Streptomyces thermoviolaceus* NBRC 13905, and *Streptomyces thermodiastaticus* JCM 4840. The high bootstrap support values (above 85–100%) confirm the robustness of the observed relationships. Comparative genomic characteristics such as genome size, G+C content, number of protein-coding genes, and biosynthetic gene cluster counts are indicated for each strain. The results suggest a strong phylogenetic and functional relationship between *Streptomyces sp.* LPB2020-019-1HS and known thermophilic species within the genus *Streptomyces*, supporting the thermotolerant nature of the studied strain.

The optimal growth temperature for *Streptomyces* sp. LPB2020-019-1HS was determined to be $55 \,^\circ$C, at which the strain exhibited the highest growth rate and sporulation efficiency. These results, together with the 16S rRNA and whole-genome phylogenetic analyses, confirm that Streptomyces sp. LPB2020-019-1HS is a thermophilic rather than merely thermotolerant strain.

### Antibiotic activity of extracts from *Streptomyces* sp. LPB2020-019-1HS

According to the experiments aimed at cultivating the strain *Streptomyces* sp. LPB2020-019-1HS on 12 nutrient media under 6 temperature conditions, we observed that the strain exhibited activity against both gram-positive and gram-negative bacteria. The maximum antibacterial activity was observed at $37\,^\circ$C against *B. subtilis* (cultivated on nutrient media SM1, NL19, SM17, SM12, SM20, and SM25), *M. smegmatis* (cultivated on nutrient media NL19 and SM17), and *E. coli*, *P. putida* and *C. glabrata* (cultivated on SM17 media). When cultivated at $28\,^\circ$C, antibacterial activity was detected against *B. subtilis* (cultivated on nutrient media SM17 and SM27Ac), *S. carnosus* (NL19 media) and *M. smegmatis* (NL19 and SM17 media). At temperatures of 45 and $55\,^\circ$C, antibacterial activity was noted against the growth of *M. smegmatis* (NL19 and SM17 media) and *B. subtilis* (NL19 and SM17 media, exclusively at $45\,^\circ$C). At cultivation temperatures of $13\,^\circ$C and $65\,^\circ$C, no antibacterial activity was observed. Table 3 lists the activities of the crude extracts. “+” indicates the presence of antibiotic activity, “-” indicates the lack of antibiotic activity.

**Table 3:** Antimicrobial activity of *Streptomyces* sp. LPB2020-019-1HS cultivated at different temperatures and nutrient media.

Nutrient media	SM1	NL19	SM17	SM12	SM20	SM24	SM25	SM27Ac	SM27N	SM27Al	R2	MM
Temperature of cultivation $28 \,^\circ$C												
* B. subtilis*	−	−	+	−	−	−	−	+	−	−	−	−
* S. carnosus*	−	+	−	−	−	−	−	−	−	−	−	−
* M. smegmatis*	−	+	+	−	−	−	−	−	−	−	−	−
* E. coli*	−	−	−	−	−	−	−	−	−	−	−	−
* P. putida*	−	−	−	−	−	−	−	−	−	−	−	−
* C. glabrata*	−	−	−	−	−	−	−	−	−	−	−	−
Temperature of cultivation $37 \,^\circ$C												
* B. subtilis*	+	+	+	+	+	−	+	−	−	−	−	−
* S. carnosus*	−	−	−	−	−	−	−	−	−	−	−	−
* M. smegmatis*	−	+	+	−	−	−	−	−	−	−	−	−
* E. coli*	−	−	+	−	−	−	−	−	−	−	−	−
* P. putida*	−	−	+	−	−	−	−	−	−	−	−	−
* C. glabrata*	−	−	+	−	−	−	−	−	−	−	−	−
Temperature of cultivation $45\,^\circ$C												
* B. subtilis*	−	+	+	−	−	−	−	−	−	−	−	−
* S. carnosus*	−	−	−	−	−	−	−	−	−	−	−	−
* M. smegmatis*	−	+	+	−	−	−	−	−	−	−	−	−
* E. coli*	−	−	−	−	−	−	−	−	−	−	−	−
* P. putida*	−	−	−	−	−	−	−	−	−	−	−	−
* C. glabrata*	−	−	−	−	−	−	−	−	−	−	−	−
Temperature of cultivation $55\,^\circ$C												
* B. subtilis*	−	−	−	−	−	−	−	−	−	−	−	−
* S. carnosus*	−	−	−	−	−	−	−	−	−	−	−	−
* M. smegmatis*	−	+	+	−	−	−	−	−	−	−	−	−
* E. coli*	−	−	−	−	−	−	−	−	−	−	−	−
* P. putida*	−	−	−	−	−	−	−	−	−	−	−	−
* C. glabrata*	−	−	−	−	−	−	−	−	−	−	−	−

### Screening of NPs produced by *Streptomyces* sp. LPB2020-019-1HS NPs related to the Nocardamine family

**Fig. 3:**
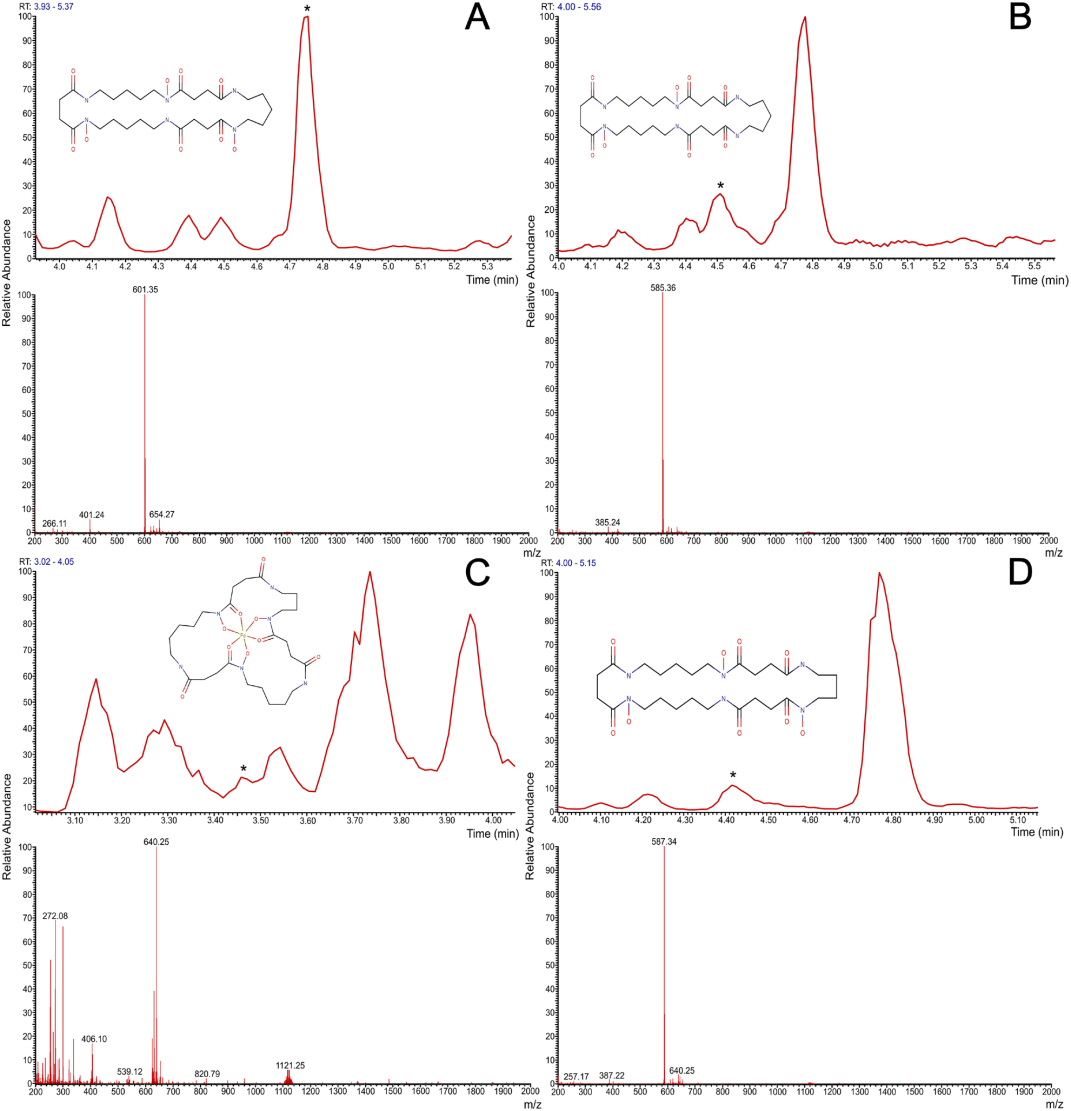
Structural formulas, mass chromatograms and mass profiles of the NPs related to the Nocardamine family produced by thermophilic *Streptomyces* sp. isolated from coldwater sponges. A – Nocardamine; B – Demethylenenocardamine; C – Ferrioxamine D2; D – Deoxynocardamine.

LC–MS is the most useful and popular technique for screening NPs. By this analysis, we found that *Streptomyces* sp. LPB2020-019-1HS synthesizes NPs related to the Nocardamine family. Thus, we detected Nocardamine (RT – 4.74 min, MM – 600.458 m/z, 4 ppm), Demethylenenocardamine (RT – 4.37 min, MM – 586.3326, 0.02 ppm), Deoxynocardamine (RT – 4.47 min, MM – 584.3510, 3.96 ppm), and Ferrioxamine D2 (RT – 2.85 min, MM – 639.2432, 1.40 ppm) (Fig.3). All the compounds were detected at $28\,^\circ$C in SM17, SM25, SM27(N), and R2 media and at $37\,^\circ$C in SM17, SM12, SM24 SM25, SM27 (AC), SM27(N), SM27(Al), and R2 media.

### Compounds potentially responsible for antimicrobial activity

This selection was conducted via multiple rounds of data sorting, identification of common and distinct peaks in mass chromatograms, and comparison of mass spectrometry data with antibiotic activity data. When the strain was cultured at $28\,^\circ$C, we observed several NPs that may be responsible for the observed antibiotic activity. The NPs presumed to be responsible for the growth inhibition of *M. smegmatis*, characterized by their retention time and mass, are presented in the Table 4 These NPSs were synthesized by the strain only in the SM17 medium and were not produced at temperatures of 13 °C and 37 °C.

**Table 4:** NPs synthesized exclusively in the SM17 medium at 28 °C and presumed to be responsible for the inhibition of *Mycobacterium smegmatis*.

Molecular mass, m/z	RT, min
705.42997	7.22
640.40384	8.34
512.34476	8.4
344.19355	9.01
494.33448	9.23
510.32938	9.23
438.27176	9.81
712.46091	9.81
496.35013	10.56
514.36057	10.56
714.47678	10.64
694.45047	11.28
492.31885	13.35
564.27121	14.06
239.22425	14.23
516.40165	16.01
613.39011	16.35
657.56913	17.8

The NPs presumed to be responsible for the activity against the growth of *B. subtilis* are presented in the Table 5. All mentioned NPs cannot be initially identified.

**Table 5:** NPs presumed to be responsible for the inhibition of* Bacillus subtilis* growth.

Molecular mass, m/z	RT, min
256.16697	5.62
238.15616	6.11
528.33982	8.87
9+ 557.33182	13.38

The list of NPs potentially associated with the antimicrobial activity of extracts obtained after cultivation at 37 °C are presented in the Table 6.

**Table 6:** NPs potentially associated with the antimicrobial activity of extracts obtained after cultivation at 37 °C.

Molecular mass, m/z	RT, min
334.0318	2.79
362.1717	6.17
404.14673	6.37
694.39011	7.4
238.19263	7.48
366.11002	8
564.19889	9.34
582.20963	9.83
610.20426	10.48
590.17771	11.62
364.09388	12.14
770.61283	12.37
338.11463	12.74
550.32743	13.02
620.18833	13.18
534.3328	13.8
546.36942	15.1
310.24994	15.58
470.3707	15.66
384.28022	15.98
342.27649	16.48
448.29697	18.38
598.41739	19.49

The NPs with an RT of 6.66 min and an MM of 422.11232 m/z were identified via the DNP database on the basis of their precise mass and biological source as Antibiotic FL 120B. In our study, activity against *B. subtilis* was demonstrated, which suggests that this antibiotic is responsible for the antimicrobial activity against *B. subtilis*.

**Fig. 4:**
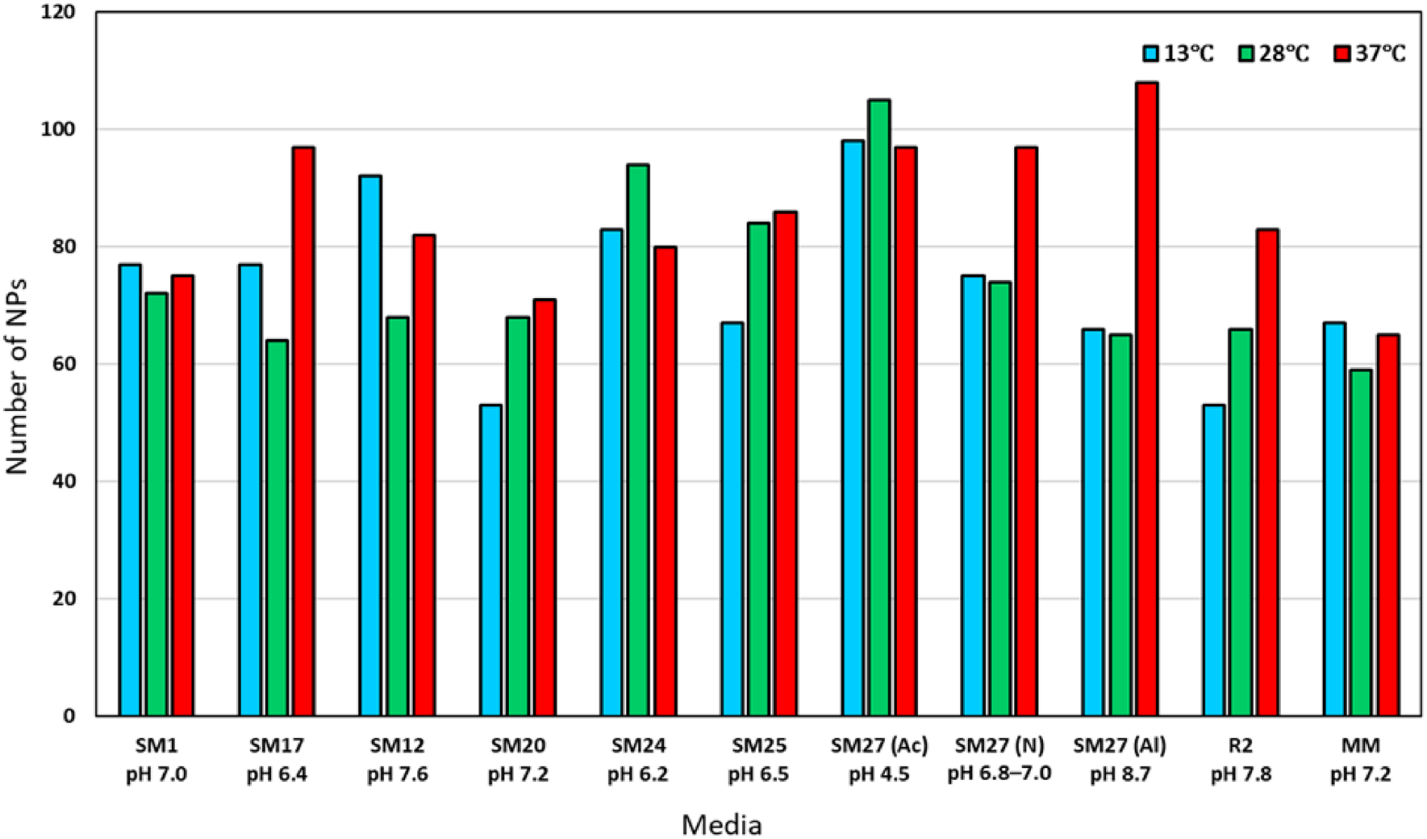
Total number of NPs produced by *Streptomyce*s sp. at different temperatures and compositions of nutrient media.

Here, we demonstrated the number of NPs produced by *Streptomyces* sp. LPB2020-019-1HS under different conditions (Fig. 4). The maximum number of NPs was detected when the plants were grown in acidified SM27 at 28 °C and alcalified with SM27 at 37 °C. The lowest number of NPs was found in SM20 and R2 at 13 °C (Fig. 4). Furthermore, when *Streptomyces* sp. was cultivated on SM1 nutrient media, minimal differences were observed in the synthesis of compounds under different temperature conditions. Cultivation of the strain in SM17, SM27(N), SM27(Al), and R2 media led to the induction of the synthesis of NPs at 37 °C.

**Fig. 5:**
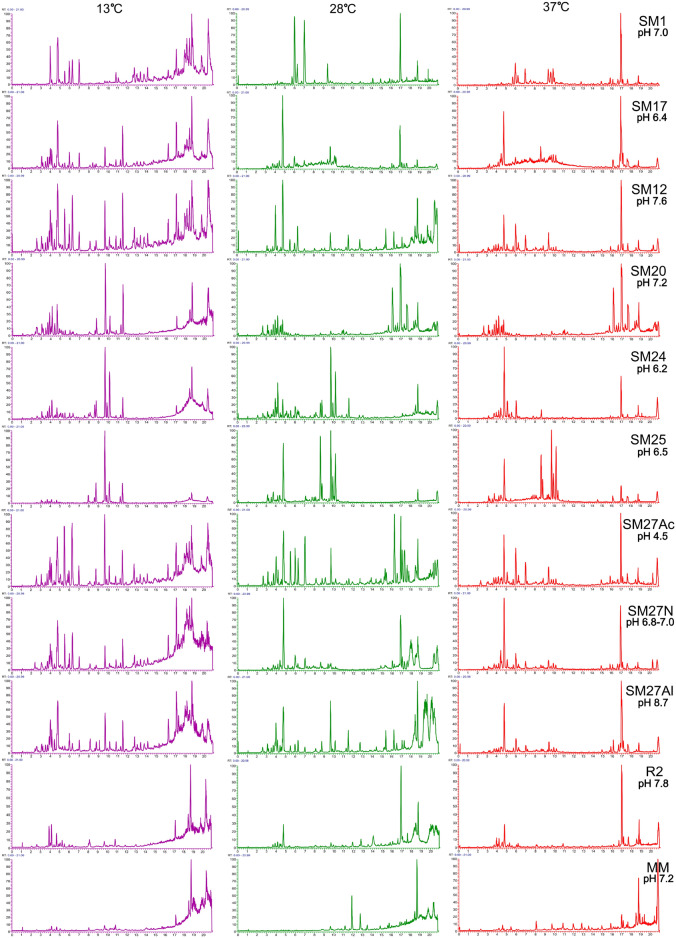
Visualized differences in mass chromatograms of extracts.

The differences in the mass chromatograms of the analysed crude extracts are shown in Fig. 5. The figure illustrates overall differences in metabolite distribution among the tested media and temperatures. Peaks are shown for qualitative comparison only; no specific compound identification is presented in this figure.

Under all temperature conditions, the thermophilic strain *Streptomyces* sp. LPB2020-019-1HS synthesized no fewer than 87 common NPs. The identified NPs with biological activity are presented in the Table 7. The biological activity of the identified metabolites was determined either according to the information available in the DNP database or, when such data were not provided in the database, based on descriptions found in relevant scientific literature sources.

**Table 7:** Identified NPs produced by *Streptomyces sp.* LPB2020-019-1HS under all temperature conditions.

Compound name	Molecular mass, m/z	ppm	RT, min	Biological activity
5-Zizaen-4-one	218.16651	2.54	10.7	Antibiotic activity
N-Methylisoleucylamic halluconylglutamic acid	452.262112	3.02	3.35	Antibiotic activity
Usabamycin	272.152478	2.05	9.06	Antibiotic activity
JBIR 120	310.13099	2.43	4.82	Antibiotic activity
Nivelactam	439.2688	7.87	12.22	Antibiotic activity
SEN 128A	234.0999	2.32	2.25	Antibiotic activity
Aggreceride B	330.27643	1.76	16.32	Antibiotic activity
Usabamycin A	272.15192	2.05	9.14	Antitumour activity^69^
JBIR 12	310.13099	2.43	4.85	growth inhibitors for cancer cells^70^
Nivelactam	439.26886	7.74	11.93	Antibiotic activity
SEN 128A	234.09978	2.83	2.29	Inhibitor of platelet agglutination
Aggreceride B	330.27631	2.12	15.85	Inhibitor of platele aggregation
Bis-(2-ethylhexyl) phthalate	390.27643	1.49	18.66	Antibiotic and antifungal activity^71^

Only at a temperature of 13 °C did the strain synthesize no fewer than 113 NPs. The identified NPs with biological activity are presented in the Table8.

**Table 8:** Identified NPs produced by *Streptomyces sp.* LPB2020-019-1HS under 13 °C.

Compound name	Molecular mass, m/z	ppm	RT, min	Biological activity
Lugdunin	782.44449	8.71	9.0	Antibiotic activity against Staphylococcus aureus^72^
INA 2770	294.18259	1.77	10.51	Antibiotic activity against Staphylococcus aureus^73^
OA 6129E	489.21347	2.08	8.41	Antibiotic activity
Megacidin	486.2468	0.62	7.02	Antibiotic activity
Penicillin N	359.11496	0.41	5.83	Antibiotic activity
Cyclo(leucylprolyl); (3S,8aS)-form	210.13611	3.42	3.87	Antitumour activity
Sarmentosamide	222.13619	2.87	7.07	Antitumour activity^74^

Only at a temperature of 28 °C did *Streptomyces* sp. LPB2020-019-1HS synthesize at least 161 NPs. The identified NPs with biological activity are presented in the Table 9.

**Table 9:** Identified NPs produced by *Streptomyces sp.* LPB2020-019-1HS under 28 °C.

Compound name	Molecular mass, m/z	ppm	RT, min	Biological activity
Butyrolactol A	526.31815	7.53	17.83	Antifungal activity
Ebelactone B	352.26062	2.1	14.52	Esterase inhibitor^75^
2-[methyl(3-phenylpropanoyl)amino]benzoic acid	283.12049	1.25	7.86	Agent against microalgae
Milbemycin B5	528.33982	9.97	8.87	Nematicidal and miticidal properties
An 88696F	362.24265	8.45	11.06	Inhibits gastric ATPase activity

Notably, at 37 °C, the thermophilic strain *Streptomyces* synthesized no fewer than 199 NPs, which were not detected at 13 °C or 28 °C. The identified NPs with biological activity are presented in the Table 10.

**Table 10:** Identified NPs produced by *Streptomyces sp.* LPB2020-019-1HS under 37 °C.

Compound name	Molecular mass, m/z	ppm	RT, min	Biological activity
Carbazomycin B	241.10959	2.0	2.22	Antibiotic activity
1-(3-hydroxy-1-azetidinyl)-7-undecene-1,5-dione; (E)-form	253.16719	2.39	7.55	Antibiotic activity
Nitrosporin	390.17798	2.84	6.79	Antibiotic activity
FL 120B	422.11222	1.94	6.66	Antibiotic activity
JI 20B	495.29502	9.27	13.88	Antibiotic activity
Dibutyl phthalate	278.15137	1.58	13.31	Antibiotic activity
Thaxtomin A; 3,3”-Dideoxy	406.16214	4.84	7.11	Phytotoxin
Thaxtomin C	392.14618	5.8	5.27	Phytotoxin
Vaccenic acid	282.25528	2.13	15.16	Algaecide

**Fig. 6:**
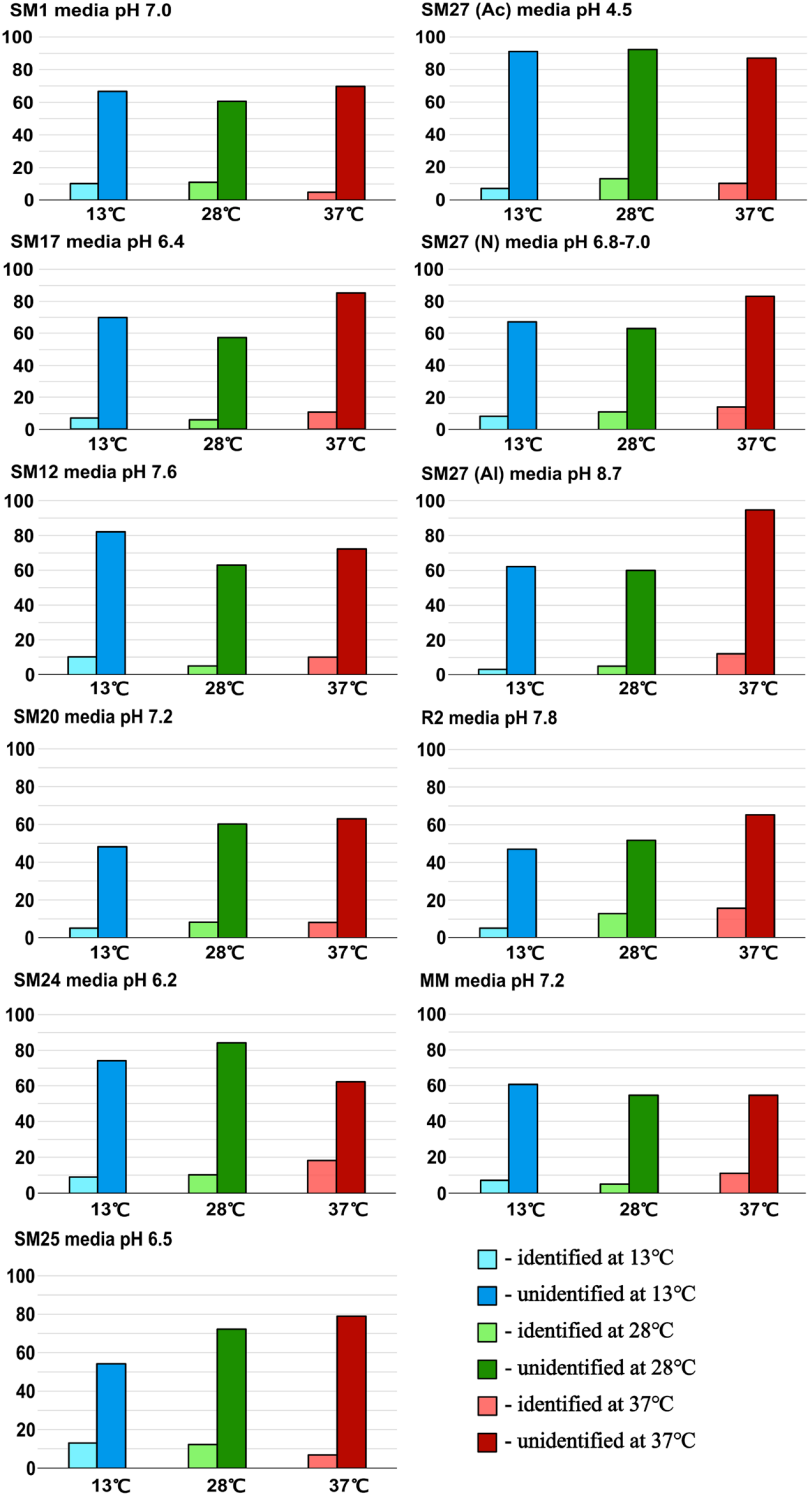
Number of identified and unidentified NPs at different temperatures and compositions of nutrient media.

Thus, the strain synthesized no fewer than 560 NPs. The number of identified and unidentified NPs synthesized under different conditions is presented in Fig. 6.

#### Genome annotation and biosynthetic gene cluster (BGC) prediction

In total, 5 860 086 reads ranging from 35 bp to 251 bp in length were generated. According to the FastQC report (v. 0.11.9), the usual read length was 250 bp, while there were very few reads with lengths of 35 bp and 251 bp. A total of 19 BGCs were identified in the genome of *Streptomyces sp.* LPB2020-019-1HS. Among them, 5 clusters showed high Similarity Confidence with known reference clusters, whereas 14 clusters demonstrated low Similarity Confidence, suggesting potential for the production of novel or structurally divergent secondary metabolites. Table 11 lists the parameters of all clusters of genes related to the biosynthesis of secondary metabolites.

**Table 11:** Secondary metabolite biosynthetic gene clusters in *Streptomyces* sp. LPB2020-019-1HS.

Locus Tag	Begin	End	Length, nt	Product/Products	Type
ctg2_120	122,434	123,492	1059	Flaviolin, 1,3,6,8-tetrahydroxynaphthalene	Type III PKS
ctg7_94	101,674	102,912	1239	Spore pigment	Type II PKS
ctg7_95	102,909	104,177	1269	Spore pigment	Type II PKS
ctg14_28	32,421	34,628	2208	Geosmin	Terpene
ctg21_68	72,082	72,486	405	Ectoine	Ectoine
ctg33_38	45,032	46,801	1770	Desferrioxamin B/Desferrioxamine E	NI-siderophore

**Fig. 7:**
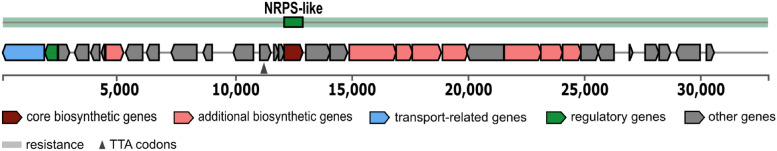
Representative picture of biosynthetic gene cluster of desferrioxamine E.

A cluster of type III polyketide synthases (T3PKSs) responsible for the synthesis of compounds such as flaviolin and 1,3,6,8-tetrahydroxynaphthalene was discovered. Additionally, a cluster of type II polyketide synthases (T2PKSs) was found. This cluster is responsible for the synthesis of kuramycin and spore pigments. A terpene cluster responsible for geosmin synthesis was also detected in the genome of the thermophilic strain. Moreover, a cluster responsible for desferrioxamine E synthesis (NRPS-like), also known as Nocardamine, was identified. A representative picture of the group of desferrioxamine E gene related to biosynthesis is presented in Fig.7.

**Fig. 8:**
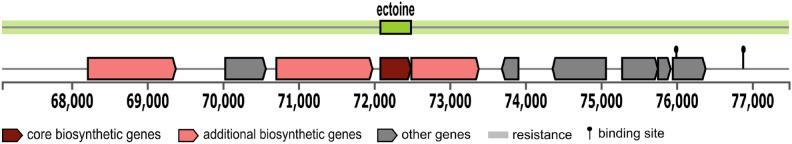
Representative picture of biosynthetic gene cluster of ectoine.

Finally, a cluster related to ectoine synthesis was also detected. A representative picture of a biosynthesis-related gene cluster of ectoine is presented in Fig.8.

## Discussion

Analysis of the metabolite profile of *Streptomyces sp.* LPB2020-019-1HS demonstrated the presence of siderophore-type compounds belonging to the nocardamine family. According to the DNP database, Nocardamine (Desferrioxamine) functions as an antioxidant, siderophore, and antibiotic and induces morphological changes in insect cells^76^. Demethylenenocardamine inhibits WNT (wingless+Int genes) signalling. WNT is an intracellular signalling pathway in animals that regulates embryogenesis, cell differentiation, and the development of malignant tumors^77^. Demethylenenocardamine and Nocardamine are siderophores produced by *Streptomyces* that sequester iron with high affinity^78^. Nocardamine is a siderophore of cyclic hydroxamic acid. Demethylenenocardamine is a lower homologue^79^. Nocardamine is a microbial siderophore that is used as a detoxification agent in cases of iron and aluminum overload due to its ability to form complexes with metals^80^. Deoxynocardamine is a siderophore in which one of the hydroxyl groups in the hydroxamic acid of the nocardamine molecule has been replaced by a hydrogen atom^80^. Ferrioxamine D2 is a growth factor and an inhibitor of protein phosphatase. Ferrioxamine D2 is a neutral compound and is comparable in its properties to ferrioxamine D1 and E^81^. Ferrioxamines represent an important and well-studied class of siderophores produced by species of *Nocardia*, *Streptomyces*, *Micromonospora*, *Pseudomonas*, and others^82^. Siderophores are low molecular weight compounds that chelate Fe3+ ions and are produced by microorganisms and plants in response to a deficiency of iron ions in the environment^83^. During the analysis of patents and inventions in the fields of medicine and pharmaceuticals, several mentions of the compound Nocardamine were identified. Patent ? CA2971824A1 (New metal complexes of nocardamine and their use in pharmaceutical compositions) describes a pharmaceutical composition containing nocardamine with at least one metal. This composition allows for modulation of cytokine levels in cells and thus provides treatment for disorders associated with inflammation. Patent ? CN102657644A (use of nocardamine in the preparation of senile dementia-resistant drugs) describes the use of nocardamine in the preparation of a drug against senile dementia. Nocardamine injections were administered in the lumbar region at a dose of 30 mg / kg of body weight for 14 days. These findings demonstrate that nocardamine injections significantly improve memory and behavior in model rats with senile dementia. However, it should be noted that, according to clinicaltrials.gov data and literary sources, there is no information on the conduct of clinical trials with these molecules. The results of this study demonstrate that the production of natural compounds by *Streptomyces sp.* LPB2020-019-1HS is significantly influenced by both the composition of the cultivation medium and the temperature regime. Such variability in the metabolic profile is consistent with the known adaptive properties of *Streptomyces* species, which are capable of modulating the spectrum of secondary metabolites in response to environmental factors. Moreover, the results of this study demonstrate the biosynthesis of natural compounds by *Streptomyces* sp. LPB2020-019-1HS is significantly influenced by both the nutrient composition of the cultivation media and the incubation temperature. This variability in the metabolic profile is consistent with the well-known adaptive properties of *Streptomyces* species, which are capable of modulating their secondary metabolite production in response to environmental factors. However, based on the genome analysis and the composition of the media used, a deeper explanation of the observed differences can be proposed. The composition of the growth medium is a key factor determining the availability of precursors for the biosynthesis of natural products. The media used in this study contained various carbon and nitrogen sources, including glucose, mannitol, maltose, glycerol, peptone, yeast and meat extracts, as well as inorganic salts. These components not only support primary metabolism and biomass accumulation but also modulate the activation of biosynthetic pathways involved in secondary metabolism. For example, siderophores of the nocardamine family-whose production was confirmed by mass spectrometry-are derived from amino acids, primarily lysine. The presence of complex nitrogen sources such as peptone and yeast extract in media such as SM17, SM12, SM25, and SM27 likely contributed to the intensive synthesis of these compounds. Sugars and sugar alcohols such as glucose, mannitol, and glycerol served as carbon sources and contributed to the construction of polyketide and peptide backbones. In media containing glucose and glycerol (e.g., SM17 and SM25), the production of antibiotics and antiparasitic compounds such as Lugdunin, INA 2770, and Penicillin N was observed. Inorganic components like NaCl, CaCO$_3$, KH$_2$PO$_4$, and MgSO$_4$ may also influence ionic balance and enzymatic activity involved in biosynthetic processes. For instance, CaCO$_3$ helps stabilize medium pH, while magnesium ions serve as cofactors in a wide range of enzymatic reactions The pH of the growth medium plays a particularly important regulatory role. A comparison of three pH variants of SM27 medium (acidic pH 4.5, neutral pH 6.8–7.0, and alkaline pH 8.7) revealed a strong correlation between pH and both the diversity and quantity of secondary metabolites. When cultivated under acidic conditions (pH 4.5) at 28 °C, more than 120 natural products were detected, including siderophores, anti-aggregation agents, and antitumor molecules. Under alkaline conditions (pH 8.7) at 37 °C, the highest total number of compounds was observed-approximately 199-including unique molecules not detected in other conditions. In contrast, the neutral pH medium supported significantly fewer secondary metabolites. These findings suggest that extreme pH conditions can act as environmental stressors that trigger the activation of specific biosynthetic gene clusters. Such activation may involve global regulatory mechanisms, including sigma factors and two-component systems, consistent with known stress-response strategies in actinobacteria. The temperature regime also had a substantial influence on the metabolic output of the strain. Notably, at 13 °C -despite low biomass and no observable antimicrobial activity in disk diffusion assays-a significant number of natural products were detected in the culture supernatant, including antibiotics and molecules with predicted antimicrobial or antiparasitic activity. This suggests that biosynthetic gene clusters remain active even under cold stress, potentially reflecting the strain’s adaptation to symbiosis with cold-water organisms such as *Lubomirskia baikalensis*. As the cultivation temperature increased to 28–37 °C, the diversity and abundance of natural products also increased, accompanied by the emergence of unique compounds and enhanced antimicrobial activity. Representatives of the genus *Streptomyces* are known for their ability to produce a wide range of biologically active secondary metabolites, including the compounds identified in this study, flaviolin, 1,3,6,8-tetrahydroxynaphthalene, spore pigment, geosmin, ectoine, desferrioxamine B and desferrioxamine E. Kuramycin is an antibiotic with a polyketide backbone made of modified or sellinic acid. Flaviolin is a product of the spontaneous oxidation of 1,3,6,8-tetrahydroxynaphthalene, which is synthesized by bacteria^84^. 1,3,6,8-Tetrahydroxynaphthalene (THN) is a secondary metabolite that serves as an aromatic polycarboxylic acid^85^. THN is commonly found in the biosynthesis of different organisms, including certain types of fungi^86^. Importantly, 1,3,6,8-tetrahydroxynaphthalene often acts as a precursor for the synthesis of complex compounds, such as melanins or other polyphenols^87^. These compounds can perform different functions in organisms, such as protection against ultraviolet radiation, antioxidant properties, and mechanisms to protect organisms from stressful situations^88^. Geosmin is a secondary metabolite with a pronounced aroma reminiscent of the smell of earth or soil^89^. This chemical compound is produced by different microorganisms, such as cyanobacteria and certain bacteria, including* Streptomyces*^90^. As noted earlier, desferrioxamine E acts as an antioxidant, antibiotic, and siderophore^91^. This molecule is widely distributed among *Streptomyce*s bacteria. Moreover, desferrioxamine E influences the growth, cell differentiation, and antibiotic synthesis of *Streptomyces* bacteria. High-resolution mass spectrometry approaches previously demonstrated that this strain synthesizes desferrioxamine E at temperatures of 28 °C and 37 °C but not at 13 °C. Ectoine is a secondary metabolite that is often synthesized by microorganisms in response to stressful conditions, such as high salt concentrations or low temperatures. Structurally, ectoine is a cyclic amino acid that plays a key role in maintaining cellular stability and protection against extreme environmental conditions. It has water-regulating properties and can help organisms survive harsh conditions by preventing protein denaturation and providing resistance to freezing. Therefore, ectoine protects cell membranes from oxidative damage and was previously described in extremophilic microorganisms^92^. These metabolites play various ecological and physiological roles, such as pigmentation, iron chelation, osmoregulation, and antibiotic or signaling activity. Phylogenetic analysis showed that *Streptomyces* sp. LPB2020-019-1HS is closely related to several thermophilic *Streptomyces* species. However, a comparative genomic analysis was limited due to the lack of annotated and published genomes for these related thermophilic strains. This reflects a larger gap in the current understanding of thermophilic *Streptomyces* biosynthetic diversity.

## Conclusion

Thus, our study was performed with a representative thermophilic strain belonging to the genus *Streptomyces* isolated from an endemic cold-water sponge in Lake Baikal. Previously, thermophilic strains were detected in hot springs in the Baikal rift zone^93–95^, such as in the Goryachinsk thermal spring^96^. However, recent data have emerged regarding the isolation of thermophilic bacteria of the genus *Thermaerobacter* from sediments in low-temperature zones on Lake Baikal^97^. This can be explained by their migration from thermal sources associated with a unique type of mud volcanism in Lake Baikal, leading to the dissociation of deep gas hydrates and excess pressure. As a result, this process provides an influx of ancient diatomic algae and thermophilic microorganisms from the lower stability limit of gas hydrates at depths of 250–300 m below the lake floor^97,98^. However, the absence of gas hydrates and thermal springs at the sampling site could reflect the anthropogenic origin of isolated actinobacteria that were caught by sponges due to their filtrating activity. These bacteria can also be carried by streams of water from deep parts of Lake Baikal due to the phenomenon of upwelling and the source of the Angara River at the sampling site. There are also data on the discovery of thermophilic microorganisms of the genus *Thermaerobacter* (*Firmicutes*) in the intestinal tract of Baikal oligochaetes^99^. Thus, sponges, as filters and symbiotic organisms, can accumulate different microorganisms with biotechnological potential. In this study, we analyzed 33 experimental cultivation conditions performed during strain isolation by thermal pretreatment at 3 cultivation temperatures and 12 liquid nutrient media. We demonstrated the synthesis of NPs belonging to the nocardamine family at 28 °C and 37 °C in different media. Furthermore, we identified several novel NPs produced by *Streptomyces* sp. LPB2020-019-1HS and highlighted the impact of both the composition of the nutrient medium and the temperature of cultivation on their biosynthetic capabilities. Maximum NP production was observed in acidified SM27 nutrient medium at 28 °C and in alcalified SM27 nutrient medium at 37 °C. The culture of the strain at 37 °C resulted in an increase in the synthesis of NP. Finally, five gene clusters related to secondary metabolism were identified. Moreover, the synthesis of metabolites belonging to the nocardamine family was confirmed by the presence of a cluster responsible for the synthesis of these compounds. Previous studies have repeatedly demonstrated that strains of *Streptomyces* sp. synthesize nocardamine^100,101^. In our previous study, we reported for the first time the synthesis of nocardamine by Baikal strains of actinobacteria^102^. Thus, despite the ultralow mineralization of Baikal water, the freshwater Actinobacteria studied have mechanisms to chelate iron ions, which are present at very low concentrations in the environment and probably make iron available to plants / mosquitoes or other symbiotic organisms^103^. Although this strain was initially classified as thermophilic based on its inhibitory activity profile and genomic affiliation, it is important to acknowledge that a full physiological assessment-particularly temperature-dependent growth rate analysis-is currently lacking. The observed antimicrobial activity at 45–55 °C may reflect either residual compound stability or limited metabolic activity under thermal stress. Nevertheless, the 16S rRNA gene sequence of *Streptomyces sp.* LPB2020-019-1HS showed 100% identity with *Streptomyces thermoviolaceus*, a known thermophilic species. Importantly, *Streptomyces thermoviolaceus* has not previously been reported in the microbial community of Lake Baikal, making this the first record of its occurrence in this unique freshwater ecosystem. This phylogenetic relationship further supports the hypothesis of a thermophilic character of the strain. We consider this distinction important and plan to perform targeted growth assays once the strain is recovered. Thus, extremophilic strains isolated from Lake Baikal are great sources of NPs, participate in ecological communication, and have great potential for biomedical developments as agents for novel drugs.

## Supplementary Information

Supplementary Information.

## Data Availability

The Whole Genome has been deposited at DDBJ/ENA/GenBank under the accession JBMFGA000000000 (BioProjec ? PRJNA1236260; BioSample ? SAMN47383545) https://www.ncbi.nlm.nih.gov/nuccore/JBMFGA000000000.
