# Supplementary material for: Genetic and biotechnological potential of thermophilic Streptomyces sp. isolated from Baikal freshwater psychrophilic sponge
Source: Sci Rep. 2025 Nov 21;15:41403. doi: 10.1038/s41598-025-25364-y (PMC12638748; doi:10.1038/s41598-025-25364-y)
Supplement: Supplementary file 1 — Supplementary Information. [file 41598_2025_25364_MOESM1_ESM.docx]

Table S1. Length of contigs generated in the Unicycler assembly. Lengths are shown in decreasing order. Supplementary 1

|  | Length (in bp) | Unicycler depth measure |
| --- | --- | --- |
| 1 | 492585 | 1.00x |
| 2 | 456542 | 0.77x |
| 3 | 384967 | 1.18x |
| 4 | 335818 | 0.73x |
| 5 | 223986 | 0.85x |
| 6 | 214603 | 1.07x |
| 7 | 191005 | 1.17x |
| 8 | 186500 | 0.70x |
| 9 | 182125 | 1.31x |
| 10 | 171352 | 0.95x |
| 11 | 169319 | 0.84x |
| 12 | 150830 | 0.88x |
| 13 | 148588 | 0.95x |
| 14 | 143147 | 0.93x |
| 15 | 139264 | 1.11x |
| 16 | 132840 | 0.81x |
| 17 | 129808 | 1.36x |
| 18 | 128617 | 0.97x |
| 19 | 126671 | 0.68x |
| 20 | 125489 | 0.72x |
| 21 | 117814 | 0.92x |
| 22 | 106791 | 1.17x |
| 23 | 101925 | 1.22x |
| 24 | 94784 | 1.18x |
| 25 | 94780 | 1.08x |
| 26 | 82305 | 0.98x |
| 27 | 79475 | 0.80x |
| 28 | 78869 | 1.31x |
| 29 | 74788 | 1.12x |
| 30 | 71634 | 1.28x |
| 31 | 71026 | 1.16x |
| 32 | 66246 | 0.99x |
| 33 | 62950 | 1.08x |
| 34 | 58991 | 1.06x |
| 35 | 52440 | 1.15x |
| 36 | 50729 | 0.86x |
| 37 | 49623 | 0.85x |
| 38 | 45471 | 1.24x |
| 39 | 33078 | 0.68x |
| 40 | 29304 | 1.40x |
| 41 | 24056 | 0.87x |
| 42 | 23569 | 1.05x |
| 43 | 23504 | 1.25x |
| 44 | 17556 | 1.18x |
| 45 | 16877 | 1.27x |
| 46 | 16777 | 1.04x |
| 47 | 16632 | 0.66x |
| 48 | 14646 | 0.69x |
| 49 | 13234 | 1.19x |
| 50 | 11054 | 0.88x |
| 51 | 10659 | 1.48x |
| 52 | 9143 | 0.73x |
| 53 | 9110 | 0.85x |
| 54 | 8291 | 0.86x |
| 55 | 7610 | 1.30x |
| 56 | 7204 | 1.41x |
| 57 | 6737 | 1.01x |
| 58 | 6571 | 1.26x |
| 59 | 5658 | 1.20x |
| 60 | 5386 | 7.06x |
| 61 | 5285 | 1.16x |
| 62 | 3590 | 0.68x |
| 63 | 2062 | 1.07x |
| 64 | 1691 | 1.66x |
| 65 | 1617 | 1.48x |
| 66 | 1373 | 2.19x |
| 67 | 1344 | 6.13x |
| 68 | 1185 | 6.28x |
| 69 | 1075 | 2.78x |
| 70 | 1017 | 1.56x |
| 71 | 969 | 0.82x |
| 72 | 914 | 1.05x |
| 73 | 901 | 1.44x |
| 74 | 880 | 1.12x |
| 75 | 806 | 2.01x |
| 76 | 792 | 1.87x |
| 77 | 743 | 2.76x |
| 78 | 735 | 5.79x |
| 79 | 659 | 5.53x |
| 80 | 626 | 5.47x |
| 81 | 597 | 1.77x |
| 82 | 586 | 1.99x |
| 83 | 515 | 2.05x |
| 84 | 506 | 0.83x |
| 85 | 456 | 2.14x |
| 86 | 454 | 3.11x |
| 87 | 446 | 3.30x |
| 88 | 391 | 1.47x |
| 89 | 391 | 0.91x |
| 90 | 368 | 0.85x |
| 91 | 365 | 1.47x |
| 92 | 353 | 2.39x |
| 93 | 351 | 1.45x |
| 94 | 351 | 1.06x |
| 95 | 351 | 2.22x |
| 96 | 347 | 1.28x |
| 97 | 339 | 2.69x |
| 98 | 334 | 1.97x |
| 99 | 324 | 2.01x |
| 100 | 305 | 2.18x |
| 101 | 298 | 0.80x |
| 102 | 277 | 1.71x |
| 103 | 258 | 2.00x |
| 104 | 254 | 2.55x |
| 105 | 247 | 0.96x |
| 106 | 247 | 1.08x |
| 107 | 242 | 0.82x |
| 108 | 242 | 0.85x |
| 109 | 237 | 1.23x |
| 110 | 234 | 9.14x |
| 111 | 226 | 2.80x |
| 112 | 201 | 3.83x |
| 113 | 185 | 2.00x |
| 114 | 183 | 1.47x |
| 115 | 179 | 2.37x |
| 116 | 176 | 2.70x |
| 117 | 174 | 2.87x |
| 118 | 168 | 3.01x |
| 119 | 167 | 1.77x |
| 120 | 161 | 2.14x |
| 121 | 161 | 1.72x |
| 122 | 160 | 1.05x |
| 123 | 155 | 2.02x |
| 124 | 151 | 4.59x |
| 125 | 150 | 2.19x |
| 126 | 149 | 2.58x |
| 127 | 139 | 1.69x |
| 128 | 132 | 11.51x |
| 129 | 131 | 2.62x |
| 130 | 125 | 5.43x |
| 131 | 111 | 2.23x |
| 132 | 101 | 2.16x |
| TOTAL | 5953768 |  |
